# Knowledge, attitude and practice of prophylactic mastectomy among patients and relations attending a surgical outpatient clinic

**Published:** 2012-09-30

**Authors:** Adetunji Saliu Oguntola, Peter Babatunde Olaitan, Olutayo Omotoso, Ganiyu Oyediran Oseni

**Affiliations:** 1 General surgery Division, Department of Surgery, College of Health Sciences, Ladoke Akintola University of Technology, Ogbomoso, Nigeria; 2 Plastic and Reconstructive Division, Department of Surgery, College of Health Sciences, Ladoke Akintola University of Technology, Ogbomoso, Nigeria

**Keywords:** Prophylactic mastectomy, breast cancer, prevention, Surgical outpatient clinic

## Abstract

**Introduction:**

Prophylactic mastectomy (PM) is uncommon in our practice. This study documents the knowledge and attitude of patients and relation to prophylactic mastectomy.

**Methods:**

Adults attending surgical outpatient unit were interviewed. Biodata, awareness of breast cancer, and attitude towards prophylactic mastectomy were inquired about and documented.

**Results:**

Two hundred and forty eight (99 men and 149 women) were involved. Most, 75.6%, were age bracket 20-29 years and 77.2% had tertiary education. Only 26 (10.4%) of the respondents had previous history of breast diseases. 96.4% were aware of cancer of the breast while 113 (45.2%) of them were aware that breast cancer gene can be inherited from parents and 60 (24.2%) believe cancer of the breast can affect women with strong positive family history. Only 64 (25.6%) of them would agree to prophylactic mastectomy if found necessary. Reasons given for possible refusal to consent to PM include effect on beauty, (40%), psychological effect, (22.8%), non-curing of disease, (18%), possible surgical complications, (7.2%), and financial cost, (1.2%). Presence of unilateral breast cancer and high risk status constituted about 71% of suggested possible indications for PM while presence of any breast disease was suggested by only 7.3% of respondents. The profession or education of respondents did not have significance on their acceptance or rejection of PM.

**Conclusion:**

Awareness of prophylactic mastectomy is low among patients in this study. Education about breast cancer and methods of prevention need to be improved.

## Introduction

Breast cancer is the commonest malignancy among women in Nigeria [1]. The impact of these lesions on the populace is enormous as it has been observed that women afflicted are about a decade younger than their Caucasian counterparts. Prophylactic mastectomy (PM) has been observed to reduce the morbidity and mortality among patients with strong genetic predisposition to breast cancer in advanced communities [2].

Women who are at high risk of breast cancer who may wish to consider prophylactic mastectomy after weighing other preventive options for breast cancer include those with a strong family history of breast cancer (especially if the breast cancer was diagnosed among several first-degree relatives, mother or sisters, before age 50),those who have tested positive for the BRCA1 or BRCA2 gene mutations and those who have a personal history of breast cancer and are at high risk for a recurrence. Prophylactic mastectomy can be primarily bilateral or unilateral [3].

In India, an increasing number of patients are opting for cancer-side mastectomies and often contralateral prophylactic mastectomies [4]. The unilateral prophylactic mastectomy can be immediately after surgery of one breast or later to the contralateral breast. It has been observed that the use of contralateral prophylactic mastectomy for invasive breast cancer rose to about 150% from 1993 to 2003 with no evidence of plateau effects in the United State of America [5]. Bilateral prophylactic mastectomy on the other hand, and reconstruction has increased secondary to numerous medical advances [6].

The lack of information about clinical benefits of contralateral prophylactic mastectomy for women with sporadic breast cancer and the factors that influence the contralateral prophylactic mastectomy decision making process are important clinical problems and a critical area of public health concern [7].

In some cases, patients who have been diagnosed with lobular carcinoma in situ as well as those at risk of breast cancer who also have breast microcalcifications (tiny calcium deposits) or who have very dense breast tissue which makes it difficult to detect breast cancer with imaging examinations such as mammography are offered prophylactic mastectomy. Late presentation is commonly observed among our patients as the women are reluctant to permit mastectomy even in the face of malignant breast lesions leading to a poor prognosis [8]. What would be the attitude of Nigerian women on the issue of prophylactic mastectomy? Men are also known to have strong influence on their wives in the black Africans; what will be the attitude of these men to prophylactic mastectomy? This study attempted to answer these questions.

## Methods

This was a prospective study among a cohort of patients and relations attending surgical outpatient clinics of Ladoke Akintola University of Technology Teaching Hospital, Osogbo, Nigeria. Period of study was between October to December 2010. All patients and relations attending the oncology clinic and consented to take part in the study during the study period had the questionnaires administered to them. Ethical approval was obtained from Ladoke Akintola University of Technology, College of Health Sciences Institution Reviewing Board. The questionnaires were pretested within the staff of the hospital.

The questionnaires were administered and they document their biodata as well as their knowledge, attitude and beliefs about prophylactic mastectomy. Their understanding of mastectomy, whether or not they would be willing to have prophylactic mastectomy when and if indicated were also inquired about. The questions asked included finding their understanding of mastectomy, whether or not they would be willing to have prophylactic mastectomy when and if indicated. The knowledge of possible effects of prophylactic mastectomy was also inquired about. Any subject who was not a patient with breast cancer and not a direct relation of the patients were excluded from the study. All consenting adult patients and relations were included in the study. The knowledge of possible effects of this procedure was also inquired about. Data were collated and analyzed using Epi info 7 with frequencies, t-test, Chi^2^. P values

## Results

Two hundred and forty eight (99.2%) of the 250 questionnaires sent out were returned. Respondents were 99 (39.9%) men and 149 (59.6%) women. Age distribution revealed age 20-29 years with the highest frequency of 189 (75.6%) and the least was in the range 50-59 age-group.

Thirteen (5.2%) of the respondents were illiterate while 6 (2.4%) of them had only primary education, 36 (14.4%) with secondary education, 193 (77.2%) had tertiary education. There were 184 (73.6%) Christians, 55 (22%) Muslims, 8 (3.2%) Jehovah's witnesses and 1 (0.4%) traditional worshipper among the respondents. Students were the largest group with 172 (68.4%) of the respondents, while the least was farmers with 2 (0.8%) (Table 1).

**Table 1:** Bio-data of respondents

	Distribution		Distribution
**Sex**		**Professions**	
Male	99	Students	172
Female	149	Traders	24
**Total**	**248**	Farming	2
		Civil servants	20
		Professionals	28
		**Total**	**246**
**Education**		Religion	
None	9	Christianity	55
Primary	6	Islam	192
Secondary	36	Traditional	1
Tertiary	193	**Total**	**248**
**Total**	**244**		

### Knowledge of breast cancer

Only 26 (10.4%) of the respondents had history of breast disease (whether benign or malignant) while 9 (3.6%) previously had other types of malignant lesions. Most of the participants, (239 or 96.4%) were aware of cancer of the breast, 7(2.8%) were not aware and 2 (0.8%) were not sure. Sources of information about breast cancer knowledge were from the mass media in 113 (45.2%) of the respondents while 103 (41.2%) were informed by medical practitioners. Knowledge of breast cancer screening was high in 210 (84.0%) of our respondents while 37(14.8%) were not aware and 1(0.8%) was not sure. Self breast examination was the most well known method of breast cancer screening in 199 (79.6%), while 19 (7.6%) were aware of the clinical breast examination and only 1(0.4%) was aware of mammography. Two hundred and twenty eight (91.2%) of the respondents believe in the screening procedure while 8(3.2%) do not and two (0.8%) were not sure. Only 113 (45.2%) of the respondents were aware that breast cancer gene can be inherited from parents, while 103 (41.2%) of them did not think it can be inherited through gene and the rest were not sure it can be inherited through gene from parents. Only 60 (24.2%) of the respondents believe that cancer of the breast can affect a woman if there is a strong history of death below age 50 years among three or four women in a family with breast cancer while the others 188 (77.8%) do not agree it could happen.

### Prophylactic mastectomy

Both sexes tend to respond more positively to use of chemo prevention and more negatively to PM, though males tend to respond more negative to PM. There is no significant difference in preference for either as a preventive measure in both sexes. More respondents are unsure of the usage of chemoprophylaxis. Similar finding were seen for both gender and religion, but it should be noted that muslims significantly responded positively to chemoprevention (X^2^= 4.3072, p = 0.0455, Fischer exact = 0.0304)(Table 2).

**Table 2:** Religion and decisions on breast cancer prevention methods

	Breast removal before becoming diseased				
	Yes	No	Unsure	Total	
**religion**					X^2^=1.525 p = 0.3406 Fischer exact = 0.1898
Christianity	51	71	16	138	
Islam	12	25	6	43	
Traditional	1	0	0	1	
**Total**	**64**	**96**	**22**	**182**	
	**chemoprevention using Tamoxifen**				
	**Yes**	**No**	**Unsure**	**Total**	X^2^=4.3072 p = 0.0455 Fischer exact = 0.0304
Christianity	72	36	25	133	
Islam	23	3	16	42	
**Total**	**95**	**39**	**41**	**175**	

Larger percentage of respondents with tertiary education was unsure of decision to be taken if faced with indications for preventive measures against BC. No significant difference was found in the responses of either those with and below tertiary education or those with none and others. (table 4). Only 11 out of 178 respondents have previous history of breast diseases and just 5 had a positive history of any other cancers. This does not have any significant effect on their responses (Table 3).

**Table 3:** Effect of education on willingness to have prophylactic breast treatment

	**Breast removal before becoming diseased**				
	**Yes**	**No**	**Unsure**	**Total**	
**Educational level**					X^2^=10.0047 p = 0.9450 Fischer exact = 1.0000 (comp tertiary against below)
None	1	5	1	7	
Primary	2	3	1	6	
Secondary	9	13	1	23	
Tertiary	52	71	18	141	
**Total**	**64**	**96**	**22**	**182**	
**Chemoprevention using Tamoxifen**					
	**Yes**	**No**	**Unsure**	**Total**	X^2^=1.4446 P = 0.229 Fischer exact = 0.183 (comp tertiary against below)
None	3	0	5	8	
Primary	1	1	1	3	
Secondary	15	2	7	24	
Tertiary	75	34	27	136	
**Total**	**95**	**39**	**41**	**175**	

**Figure 1:**
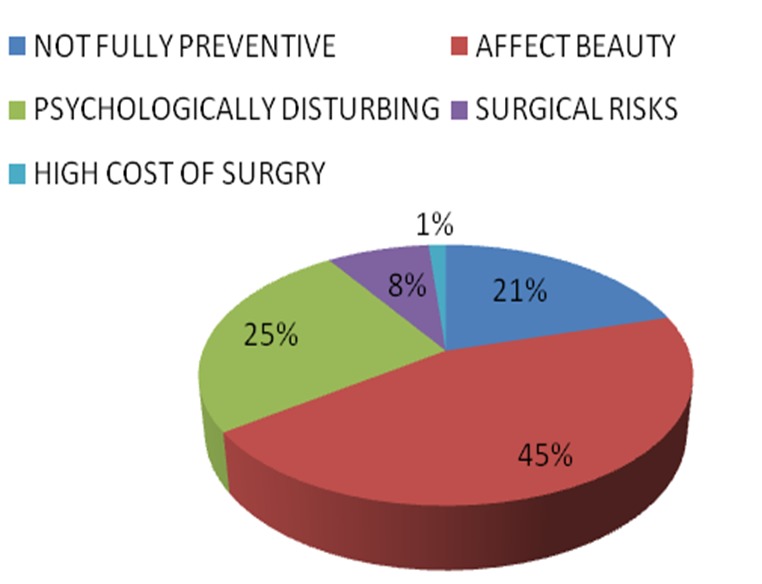
Participants’ suggested reasons for possible rejection of prophylactic mastectomy

**Figure 2:**
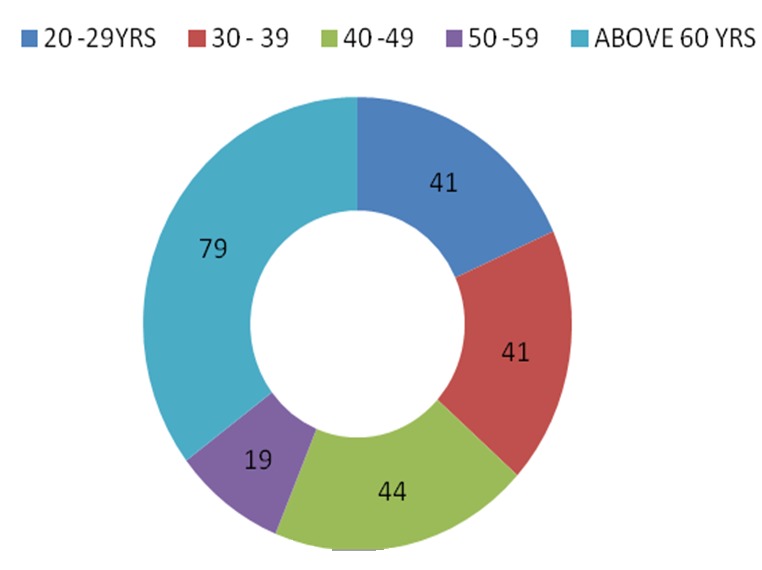
Participants’ suggested possible age range for mastectomy

**Figure 3:**
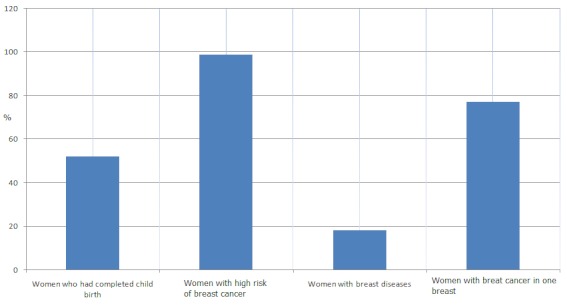
Participants’ suggested condition of women who should have prophylactic mastectomy

When asked if they would accept prophylactic mastectomy if indicated, Only 64 (25.6%) of the respondents would agree to prophylactic mastectomy if found necessary, while 97 (38.8%) would not and others 87 (35.1%) were not too sure of the decision would be to this approach to breast cancer prevention. Reasons given for possible refusal to consent to prophylactic mastectomy include effect on beauty, (40%), psychological effect (22.8%), non-curing of disease, (18%), possible surgical complications, (7.2%) and financial cost (1.2%) (Figure 1). Largest percentage of the respondents (35.3%) suggested that PM should only be done above the age of 60 years while less than 40% suggested age less than 40yrs (Figure 2). Presence of unilateral breast cancer and high risk status constituted about 71% of suggested indications for PM while presence of any breast disease was suggested by only 7.3% of respondents (Figure 3). The profession of respondents did not have significance on their acceptance or rejection of PM.

## Discussion

Breast cancer is the leading female malignancy in Nigeria [1]. Although, patients in communities with high level of awareness usually present with less advanced stages of breast cancer as a result of adoption of screening methods [9, 10] those in communities with low level of awareness often present late. In Nigeria, about two-third of patients with this disease present with advanced stages of lesion when therapy offers minimal benefit [8]. Current strategies in use for reducing the risks of breast cancers in high risk individuals include regular surveillance, chemoprevention, prophylactic mastectomy and or oophorectomy. The risk reduction potential for bilateral oophorectomy may be higher in a BRCA1 &2 carrier who is known to be at a higher risk of both ovarian and breast cancers [11].

Knowledge and attitude of patients in this environment about prophylactic mastectomy with subsequent reconstruction is therefore sought to plan for education of the community on breast cancer prevention through prophylactic mastectomy and breast reconstruction. Prophylactic mastectomy is a rare practice in our environment.

The number of prophylactic mastectomies (PM) in the United States has recently increased due to a better understanding of the genetic and biological behavior of breast cancer [1]. Bilateral prophylactic mastectomy is associated with a reduction in the risk of breast cancer by as much as 90% among women with an increased risk of breast cancer due to a strong family history of breast cancer. Because of the physical and psychological effects of bilateral mastectomy and the irreversibility of the procedure, decisions regarding this option must be carefully considered on an individual basis in association with risk assessment and counselling [11].

Selected high-risk women without breast cancer choose to undergo bilateral prophylactic mastectomy (BPM) to reduce their risk of developing the disease. Several studies have reported that BPM significantly reduces, but does not eliminate breast cancer risk [12]. Women with breast cancer are electing for contralateral prophylactic mastectomy (CPM) to reduce the risk of developing contralateral breast cancer [13]. In advanced centres, the demand for bilateral mastectomy and immediate breast reconstruction has increased over the last years, primarily due to the development of genetic testing [14]. It has been done in a number of cases of Cowden syndrome (CS), a rare, autosomal dominant inherited disorder associated with multiple benign and malignant neoplasms, including breast cancer [15] and families with an increased risk for breast/ovarian [16]. Rates of bilateral prophylactic mastectomy and contralateral prophylactic mastectomy have increased over the past decade with bilateral micro-vascular breast reconstruction playing an increasing role in breast cancer care [17] and Patients generally expressed a preference for autologous material for breast reconstruction and an excellent aesthetic result [18].

This study revealed a high level of awareness of cancer of the breast among the respondents, information obtained being mainly from medical practitioners. Knowledge of the breast cancer screening was also found to be high but only one out of four respondents would agree to prophylactic removal of their breast, this is rather low. The most common reason given by respondents for rejection of prophylactic mastectomy is the claim that it would affect their beauty, other reasons such as psychological effect, not completely preventable, possible surgical complications for a disease that is yet to and may never occur as well as the financial cost of the procedure in the face of other financial needs and challenges. It has been reported to affect the quality of life [19] while Bradberg Y et al [20] also reported a substantial proportion of the women reported problems with body image e.g., self consciousness, feeling less sexually attractive, dissatisfaction with the scars, and reduced sexual pleasure 1-year post-BPM as compared with before operation. The respondents in this study tends to prefer chemoprevention to surgical prevention, this is in keeping with the findings HT See et al [21] who assessed the view of the general public in Singapore on cancer prophylaxis. This would not be unconnected with the perceived fear of complications associated with operations in general and cosmetic in relation to breast operations in particular. Age, education, and profession do not have any significant impact on the willingness to allow prophylactic mastectomy. This is different from a study from India where the age of the patients, family history of breast cancer fewer children positively influence prophylactic mastectomy [4].

An important limitation to this study is the generally low level of knowledge of prophylactic mastectomy in our environment as this is rarely practiced and patients and relation may have difficulty understanding what prophylactic mastectomy is all about. The mass media has played a role in informing the public about breast cancer from the response of the subjects in this study. Their role in educating the people more about the impact of prophylactic mastectomy, indications for it and analysis of data from other parts of the world on the progress made with this option of managing such patients should be done.

## Conclusion

There is good awareness about breast cancer but poor knowledge of prophylactic mastectomy among the subjects. Only 25% will accept prophylactic mastectomy when indicated. Presence of and high risk of breast cancer were most suggested as indications for prophylactic mastectomy. Health workers are therefore implored to educate patients and relations about breast cancer and prophylactic mastectomy.
